# Unraveling Atopic Dermatitis: Insights into Pathophysiology, Therapeutic Advances, and Future Perspectives

**DOI:** 10.3390/cells13050425

**Published:** 2024-02-28

**Authors:** Ashutosh Pareek, Lipika Kumari, Aaushi Pareek, Simran Chaudhary, Yashumati Ratan, Pracheta Janmeda, Sanam Chuturgoon, Anil Chuturgoon

**Affiliations:** 1 Department of Pharmacy, Banasthali Vidyapith, Banasthali 304022, India; aayushipareek26@gmail.com (A.P.); simranchaudhary1015@gmail.com (S.C.); yashu.bu@gmail.com (Y.R.); 2 Department of Bioscience and Biotechnology, Banasthali Vidyapith, Banasthali 304022, India; lipikals007@gmail.com (L.K.);; 3 Northdale Hospital, Department of Health, Pietermaritzburg 3200, South Africa; 4 Discipline of Medical Biochemistry, School of Laboratory Medicine and Medical Sciences, University of KwaZulu-Natal, Durban 4041, South Africa

**Keywords:** atopic dermatitis, therapeutics, interleukins, interferon, T helper cells, Janus kinase inhibitors

## Abstract

Atopic dermatitis (AD) is an inflammatory skin condition that frequently develops before the onset of allergic rhinitis or asthma. More than 10% of children are affected by this serious skin condition, which is painful for the sufferers. Recent research has connected the environment, genetics, the skin barrier, drugs, psychological factors, and the immune system to the onset and severity of AD. The causes and consequences of AD and its cellular and molecular origins are reviewed in this paper. The exploration of interleukins and their influence on the immunological pathway in AD has been facilitated by using relevant biomarkers in clinical trials. This approach enables the identification of novel therapeutic modalities, fostering the potential for targeted translational research within the realm of personalized medicine. This review focuses on AD’s pathophysiology and the ever-changing therapeutic landscape. Beyond the plethora of biologic medications in various stages of approval or development, a range of non-biologic targeted therapies, specifically small molecules, have emerged. These include Janus kinase (*JAK*) inhibitors like Baricitinib, Upadacitinib, and Abrocitinib, thus expanding the spectrum of therapeutic options. This review also addresses the latest clinical efficacy data and elucidates the scientific rationale behind each targeted treatment for atopic dermatitis.

## 1. Introduction

Atopic dermatitis (AD), also known as eczema, is a prevalent autoimmune skin disease affecting people worldwide. The common symptoms of this disease are skin irritation and redness. Eczematous skin lesions are the hallmark of AD, an itch-inducing, inflammatory skin disorder. AD is characterized by elevated levels of skin eosinophils, neutrophils, and mast cells; lymphocyte infiltration; and increased immunoglobulin (Ig) E levels [1]. Itching, rash, bleeding, and erosions are also present. About 80% of AD cases typically initiate during infancy or childhood, while the remaining cases emerge in adulthood [2]. AD has a highly heterogeneous natural history, with localized or widespread eczematous lesions and sensitive, dry skin, and it is commonly accompanied by pruritus [3]. Age, weight, and ethnicity all affect heterogeneous clinical AD manifestations differently, with significant effects on patients and their quality of life (socioeconomic aspect), with huge financial investments every year [4].

Typically, AD causes plaques and itchiness. These plaques frequently affect the elbows, knees, face, neck, and wrists. The skin may itch and exude a clear fluid if the plaque is scratched. Over time, frequent scratching or pulling might thicken the skin in the affected area [5]. Although this disease can affect people of any age, AD is an inflammatory condition primarily affecting children.

Children suffering from AD may have a risk of developing asthma, allergic rhinitis, food allergies, and rheumatoid arthritis and a 75% risk of developing the common cold [6]. Recent studies have identified cardiovascular and neuropsychiatric disorders as significant comorbidities associated with AD, yet the underlying biological mechanisms linking these conditions to AD are still not fully understood [7,8].

AD is categorized into three distinct subsets, differentiated by the age at which the condition first appears. Each subset has unique features:

- Infantile (with an onset below two years)—In this case, the patient presents acute lesions characterized by poorly defined redness (erythema) accompanied by swelling (edema), small blisters (vesicles), scratch marks (excoriations), and a clear fluid discharge (serous exudate) on the face, cheeks, and scalp.
- Childhood (seen from two years to adolescence)—In this case, the nature of eczema tends to evolve into a more localized and chronic condition. The acute, widespread erythema and vesicular lesions of infancy give way to paler redness, increased dryness (xerosis), and less-distinct lesions. These changes predominantly affect the body’s flexural areas, such as the inner elbows and knees, where the skin may also thicken (lichenify) due to chronic scratching.
- Adult (after puberty)—This form manifests as chronic hand eczema, exclusively affecting the hands, or it may also involve head–neck dermatitis, impacting the upper trunk, shoulders, and scalp [9].

This review focuses on elucidating the pathophysiological mechanisms underlying the development of AD and identifies potential targets for pharmacological intervention. Additionally, it explores recent advancements in the management of AD, shedding light on innovative approaches and strategies.

## 2. Pathogenesis of AD

The pathogenesis of AD is multifaceted, involving genetic predispositions, abnormalities in skin function, immune system dysregulation, and environmental influences [10]. The essential membrane protein filaggrin (*FLG*) has been discovered to have the greatest genetic association with AD [11].

Dephosphorylation and proteolytic cleavage transform Pro-*FLG* polymers into *FLG* monomers, crucial for stratum corneum (SC) formation and keratin buildup. *FLG* null mutations weaken the skin barrier and heighten AD risk. Having *FLG* null alleles may trigger early AD onset, potentially extending into adulthood. In addition to *FLG* gene mutations, factors like DNA methylation, *FLG* copy number variations, environmental influences (skin irritation, damage, and low humidity), cytokines (Th2, interleukins (*IL*)-*17*, *IL-22*, *IL-25*, and *IL-31*) reducing *FLG* expression, skin microorganisms, and treatments (topical and systemic) can also alter *FLG* levels [11,12]. Given that *FLG* plays several roles in the development and progression of AD, it is possible that decreased levels of any of the components of the differentiation of the epidermal complex could significantly impact how well the epidermal barrier operates as an entire system [12]. While the lack of agreement on the role of *FLG* does not completely eliminate the possibility of other host-related factors causing AD, it does suggest that environmental factors play a significant role universally. Factors like exposure to pollution and the excessive use of soap can weaken the skin’s microbial barrier, potentially leading to AD development [13]. While the multifaceted immunological etiology of AD has been subjected to extensive scrutiny, the existing heterogeneity across diverse populations remains an area requiring more in-depth exploration. This emphasizes the imperative for personalized treatment approaches, recognizing the intricate immunological complexity inherent in this disease [14]. The cells involved in host–environment interaction resulting in skin inflammation are eosinophils, basophils, dendritic cells (DCs), keratinocytes, mast cells, macrophages, and type 2 innate lymphoid cells (*ILC2s*). Additionally, reductions in the levels of epidermal barrier proteins, such as *FLG*, spinous layer proteins (*SPINK*), and claudins, alongside alterations of antimicrobial peptides, play a significant role [15]. 

**Figure 1:**
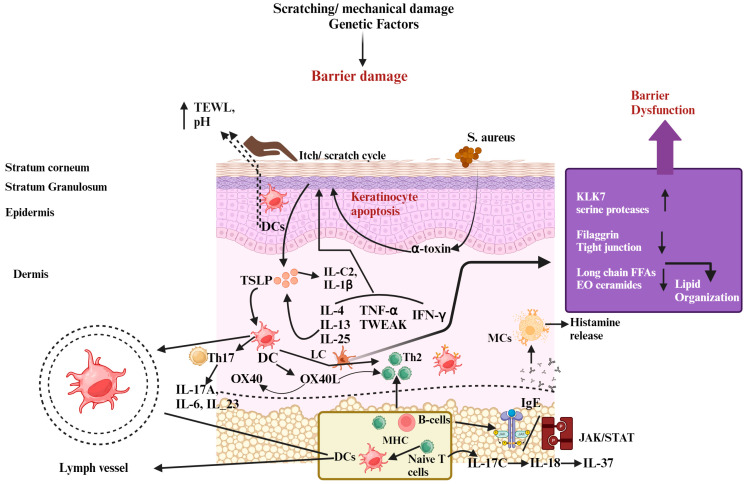
The pathophysiology of atopic dermatitis (AD) involves several factors. Skin barrier dysfunction, exposure to allergens, environmental influences, and imbalances in the skin–gut microbiota all contribute to AD development. Additionally, mutations in the filaggrin gene (*FLG*) lead to increased trans-epidermal water loss (TEWL), higher pH levels, and disrupted production of peptides that defend against infections, all of which weaken the skin barrier. This impaired barrier makes it easier for allergens to enter the skin and promotes colonization by *Staphylococcus aureus*, which forms biofilms and produces harmful substances that trigger mast cell degranulation, releasing inflammatory compounds like histamine and contributing to elevated IgE levels. Furthermore, after the skin barrier is disrupted, keratinocytes produce immune-regulating molecules such as *IL-1β*, *IL-25*, *IL-33*, and *TSLP*, which activate immune responses involving Th2, Th17, and Th22 cells. Th2 cells release cytokines that further disrupt barrier function by reducing the expression of *FLG*. The itch–scratch cycle worsens due to mast cell degranulation and amplified sensory nerve signals, aggravating barrier dysfunction. Recent studies also suggest the involvement of Th17-related cytokines in AD development. Moreover, a leaky gut resulting from gut dysbiosis allows metabolites and inflammatory substances to reach the skin, triggering a strong Th2 immune response and causing significant tissue damage. Abbreviations: *KLK-7*, kallikrein-7; IL, interleukin; *IFN-γ*, interferon-gamma; *TSLP*, thymic stromal lymphopoietin; TEWL, trans-epidermal water loss; *JAK*, Janus kinase; *STAT*, signal transducers and activator of transcription.; Th, helper cell; DC, dendritic cells; B-cell, bone-marrow cell; IgE, Immunoglobulin E; *TNF-α*, tumor necrosis factor alpha; LC, Langerhans cells; MHC, major histocompatibility complex; MCs, mast cells. Symbols: ↓, decrease; ↑, increase.

Both T and B cells, together with their respective cytokines, contribute to this disease’s immunological profile, highlighted by a dominant Th2 axis (including *IL-4*, *IL-13*, *IL-5*, thymic stromal lymphopoietin (*TSLP*), and *IL-31*), elevated Th17/*IL-23* and Th22 pathways, and increased IgE levels. Furthermore, changes in microbial diversity on the skin, characterized by an excessive presence of *S. aureus* strains, highlight the complexity of AD’s etiology [15]. The main connection between skin bacteria and AD is *S. aureus*, often found on the skin of AD patients [16]. Skin problems deriving from AD make it easier for *S. aureus* to grow [17]. When there is a lack of *FLG*, there is usually more *S. aureus* in the skin’s microbiome [18]. *S. aureus* prompts keratinocytes to produce proteases, worsening the skin’s barrier [19]. It also releases harmful substances like δ-toxin and α-toxin; δ-toxin can trigger mast cells to release substances without killing them, particularly when IgE is present [20]. In mice, *S. aureus* strains with δ-toxin caused more skin inflammation compared to those lacking it. [21] When mast cells are activated, they release various substances linked to inflammation. These include cytokines associated with Th17 cells like *IL-6*, *IL-17A*, and *IL-23*, as well as proinflammatory cytokines such as *IL-1β*, *IL-6*, and *IL-8*, and chemokines like macrophage inflammatory protein (*MIP*)-*1α*, *MIP*-*1β*, and Monocyte chemoattractant protein (*MCP*)*-1* [22,23]. These substances, combined with mediators from Th1 cells like *IL-1β*, *IL-6*, *IL-8*, *IL-10*, and interferon (*IFN*)-*γ*, initiate the ongoing inflammation seen in AD [22,23]. The Th2 cytokines *IL-4*, *IL-5*, and *IL-13* play a pivotal role in AD and have been linked to increased IgE response and eosinophils in AD patients. In AD patients, *IL-4* and *IL-4* receptors on peripheral blood lymphocytes were aberrant, with an increased production of *IL-13* [14]. It was previously demonstrated that AD patients lost their capacity to release *IFN-γ* in response to different stimuli. Researchers have found a notable connection wherein the amount of *IFN-γ* produced is linked to lower levels of IgE found in the blood of individuals with AD. Studies on patients with acute AD revealed that they had higher amounts of *IFN-γ* and *IL-4* in the skin and peripheral blood. These cytokines are secreted by T cells that are specific to the allergens [24]. When an antigen is encountered by antigen-presenting cells such as DCs, they release *TSLP* and initiate signaling pathways that activate naïve T cells. This leads to the differentiation of T cells into Th1 and Th2 subtypes, which release interleukins and chemokines to combat the antigen. However, this immune response contributes to inflammation and may exacerbate skin barrier dysfunction in AD rather than directly cause barrier penetration (Figure 1) [25].

**Figure 2:**
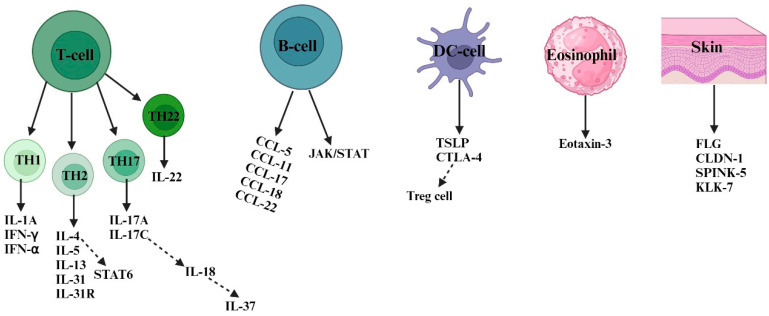
Numerous susceptibility factors and their cells of origin are involved in atopic dermatitis. In response to invading allergens and antigens, skin-resident DCs and keratinocyte cells (*FLG*, *CLDN-1*, *SPINK-5*, and *KLK-7*) release inflammatory cytokines, including *TSLP* and *CTLA4* as well as chemokines, to attract other types of immune cells, such as B-cells, eosinophils, and T cells. In AD skin, there is a coexistence of Th1, Th2, Th17, and Th22 responses. However, Th2-type responses play a predominant role in the pathogenesis of AD. Th2 cells mediate type 2 skin inflammation by secreting cytokines like *IL-4, IL-5*, *IL-13*, and *IL-31* and their receptors. Both acute and chronic AD involve Th1, Th2, Th17, and Th22 responses. In chronic AD, Th1 activation is supported by proinflammatory cytokines (such as *IL-1A*, *IFN-α*, and *IFN-γ*) secreted by skin dendritic cells. Th2 cells induce keratinocyte apoptosis via *STAT 6* secretion, while Th22 cells promote skin remodeling and thickness through *IL-22* secretion. B-cell-type responses also contribute to AD pathogenesis by secreting chemokines (such as *CCL*-*5*, *CCL*-*11*, *CCL*-*17*, *CCL*-*18*, and *CCL*-*22*) that activate the *JAK/STAT* pathway. Th17 cells release inflammatory cytokines like *IL-17A* and *IL-17C*, which further activate other cytokines (such as *IL-18* and *IL-37*) responsible for AD inflammation. Abbreviations: *FLG*, filaggrin; *CLDN-1*, claudin 1; *SPINK5*, serine protease inhibitor; *KLK*-*7*, kallikrein-7; IL, interleukin; *IFN-α*, interferon alpha; *IFN-γ*, interferon-gamma; *CCL*, chemokine C-motif ligand; *CTLA-4*, cytotoxic T-lymphocyte-associated antigen-4; *TSLP*, thymic stromal lymphopoietin; *JAK*, Janus kinase; *STAT*, signal transducers and activator of transcription.

A soluble component known as *TSLP*, which promotes Th2 responses, increases in quantity in AD skin. The Th2-specific cytokines *IL-4* and *IL-13*, as well as *IL-25* and *IL-33*, are included in the soluble factors. These factors have an impact on keratinocyte activity and skin integrity (Figure 2). Excessive Th2 cytokine production on AD skin triggers the serine protease kallikrein 7 (*KLK7*), and high serine protease levels are detrimental to the skin [26]. *IL-9*, *IL-10*, *IL-17*, *IL-22*, *IL-31*, *IL-33*, signal transducer and activator of transcription 6 (*STAT 6*), and cytotoxic T-lymphocyte-associated protein 4 (*CTLA4*) are genes that are responsible for AD development. Chemokines, including chemokine ligand (*CCL*)-*22*, *CCL18*, *CCL17*, *CCL11*, and *CCL5*, ascend in the skin during inflammation and promote the attraction of inflammatory T cells and DC subtypes [27]. Various types of immune cells work together in specific locations and at specific times in the inflamed skin of AD patients. Understanding how they interact on different levels—organic, cellular, and molecular—will aid in the development of novel treatment methods [28].

## 3. Emerging Therapies and Therapeutics

AD pathophysiology has been comprehensively discussed. Data on the pathogenesis of AD show that so far, research on AD exhibits a wide range of treatment products representing the field of prevention, which includes environmental and exosomal factors (like a westernized lifestyle, food diversity, humidity, water hardness, etc.) [29,30], followed by the skin microbiome [31], the epidermal barrier [32], the immune and inflammatory response, and the itch-circulatory response [33]. These interactions take place in unidentified genetic and epigenetic contexts. Along with the effects of the inflammatory response, such as the involvement of other organs, this is accompanied by immune system modifications. Additionally, as AD involves complicated immune pathways, there is potential to investigate bispecific or trispecific antibodies for treatment [34]. The management of the disorder varies based on the unpredictable nature of each patient’s condition and has two primary objectives: the prompt and effective treatment of acute flare-ups and the more challenging task of controlling the disease over the long term. Therefore, in addition to effectiveness, assessing the long-term safety of any new compound in a clinical development program is crucial [34]. 

### 3.1. Microbiome Modulation

Altering microbiota diversity helps in modulating the development of AD. Different strategies have been proposed for the modulation of the skin’s microbiome. Two clinical trial products (MSB-01 and DB-001) are currently being used to investigate microbiome transplantation and bacterial modulation [35]. However, there are currently no conclusive research findings supporting this theory. The skin microbiome of AD patients has a greater abundance of Gram-negative bacteria in the *Rosemonas mucosa* membranes as compared to healthy skin, which is more composed of Gram-positive bacteria [36]. A mucosal erythrocyte series (FB-401), whose therapeutic capabilities may include activating tissue healing and inhibiting Toll-like receptor 5 (*TLR5*) signaling and tumor necrosis factor receptor (*TNFR*), has been developed [37]. The topical treatment of *Staphylococcus hominis* A9 (ShA9), involving the use of a similar bacteriotherapeutic approach, kills *S. aureus* and inhibits *S. aureus*-produced toxins, allowing the microbiome to recover [38]. The bacterium *Nitrosomonas eutropha* (B244), which can produce nitric oxide by oxidizing ammonia, has been utilized to treat AD [39]. A synthetic antimicrobial cationic peptide called omeganan pentachloride (CLS-001) is now undergoing clinical testing as a possible topical treatment for managing dysbiosis [40]. Several microbiome modifiers designed for oral use are currently in phase I trials, including EDP1815, STMC-103H, and KBL69 [41]. Future strategies are likely to focus on gaining a better understanding of how bacterial quorum sensing and host immune responses influence the modulation of the skin microbiome. It may be possible to use interventions targeting the skin microbiome, possibly during infancy, to address this disease at an early stage. Determining the most effective timing for intervention could be crucial for enhancing disease management and potentially restoring a healthy adaptive immune response against *S. aureus* [42].

### 3.2. Targeting the Function of the Epidermal Barrier

There are two strategies aimed at restoring the epidermal barrier function in AD. The first involves developing products that specifically target the biochemical changes associated with this condition. However, the lack of understanding regarding the functional genetics of the various structures involved presents a significant obstacle to this approach. The second strategy focuses on effectively managing the underlying inflammatory response, although this may not completely restore barrier function. As a result, the current approach involves individualized and empirically adapted skincare using emollients or moisturizers alongside inflammation control [43,44]. This combined approach remains the primary method for improving barrier function, alleviating dryness, and reducing water loss and is considered fundamental therapy [43]. Supporting this idea, even basic products like petrolatum have been found to enhance antimicrobial activity and improve epidermal barrier function [44]. While initial studies showed promise in using emollients and moisturizers to prevent AD in high-risk newborns, a recent report has raised doubts about the effectiveness of this strategy [45].

### 3.3. Controlling Immunological Response

**Figure 3:**
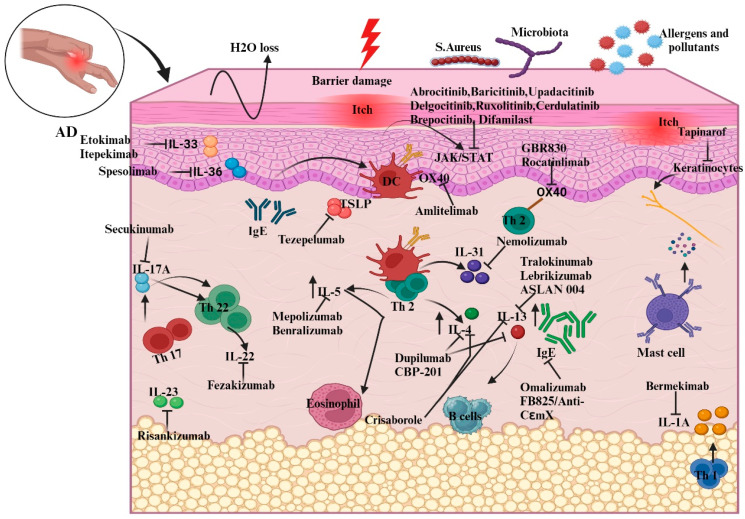
Illustration of new biologic targets in AD pathogenesis and systemic treatments guiding the immune response. The figure outlines how these therapies adjust interleukin and chemokine activity in AD, highlighting cytokine modulation by specific drugs. For instance, etokimab targets *IL-33* overexpression, while dupilumab, a monoclonal antibody, decreases elevated *IL-4* levels. Abbreviations: IL, Interleukin; Th, helper cell; DC, dendritic cells; *TSLP*, thymic stromal lymphopoietin; B-cell, bone-marrow cell; IgE, Immunoglobulin E; *JAK*, Janus kinase; *STAT*, signal transducer and activator of transcription.

Two kinds of responses are involved in AD pathophysiology: innate and adaptive immunological responses. Controlling these responses using several measures or related therapeutics (Table 1) helps to ameliorate the infiltration of AD (Figure 3).

#### 3.3.1. Innate Immune Response

The role of the innate immune system was demonstrated using animal models in the early phase of AD [46].

- Aryl-hydrocarbon receptor (*AhR*)

A ligand-activated transcriptional factor, known as the *AhR*, plays a dual role in the development of numerous types of skin inflammation, including AD [47]. *AhR* can be an excellent option for a pharmacological strategy as it involves keratinocyte expression and epidermal DCs’ residence. Immunohistochemistry and transcriptome studies have shown that coal tar binds to *AhR* and restores *FLG* expression [48]. 

Tapinarof (benvitimod), which is a natural agonist of *AhR*, decreases inflammatory reactions when topically administered in both animal models and human skin. The most common adverse events were upper-respiratory-tract infections and folliculitis. The data indicate that this compound shows potential as a new and promising choice for treating AD and psoriasis, both chronic inflammatory skin conditions [49].

- SPINK5

*SPINK5* is mostly prevalent in keratinocytes and is associated with skin differentiation. When inflammation occurs, it infiltrates *TSLP*, *IL-33*, and *IL-25*, activating Th2 cells [50]. There are possible therapeutic targets that include these interleukins. Th2 activation is enhanced by the cytokine *TSLP*, which is produced in response to pro-inflammatory stimuli. These stimuli promote the production of *IL-4*, *IL-5*, *IL-13*, and *TNF-α*, affecting mast cells, DCs, and natural killer cells, among other immune cells. In patients with acute or chronic AD, keratinocytes exhibit elevated expression of *TSLP* [46,51]. 

Patients with chronic and unmanageable allergic asthma responded favorably to the anti-*TSLP* antibody tezepelumab (AMG 157) [52,53]. In contrast, a phase IIa trial involving patients with AD revealed that 64.7% of patients achieved the EASI50 endpoint, which can be compared to 48.2% in the placebo group. However, interpreting these results is challenging because all the patients were permitted to use topical corticosteroids (TCSs) [54].

- IL-36R

*IL-36* is part of the innate immune system and is increased in the skin of both psoriasis and AD patients. Notably, in mice, infection with *S. aureus* leads to inflammation that relies on *IL-36R* and *IL-1R*. As a result, a treatment called spesolimab, which targets *IL-36R* and has been effective in treating a rare type of psoriasis, is being studied in a phase-II trial involving 51 AD patients [55]. 

It is still unclear which specific pathways within the innate immune system play a significant role in the early stages of AD in infancy and throughout the progression of this disorder. Understanding this aspect is essential for developing effective targeted treatments [42].

- IL-1

*IL-1*, a crucial cytokine for innate immunity, induces inflammation. *IL-1R* and *IL-1* are both highly produced in skin cells and need to be balanced for epidermal homeostasis to be maintained [56]. *IL-1* expression is raised in patients with inflammatory cutaneous diseases such as psoriasis, alopecia areata, and AD. Through an increase in Th1 expression, *IL-1* leads to the formation of Th17 and Th2 cells as well as the chronification of AD lesions [57]. 

Phase-II, open-label, dose-escalation trials have been conducted on the anti-*IL-1* monoclonal antibody bermekimab (MABP1). The corresponding study compared two groups receiving different doses of bermekimab: 200 mg and 400 mg. The 400 mg group showed greater improvements in Eczema Area and Severity Index (EASI) scores, along with other severity measures like IGA, pain, and itchiness, compared to the 200 mg group [58].

- IL-33

In AD, keratinocytes release *IL-33*, which causes the skin barrier to deteriorate and become inflamed; when the skin is exposed to allergens or staphylococcal toxins, it generates excessive amounts of *IL-33*. *IL-33* activates Th2 responses and increases the production of *IL-4*, *IL-5*, and *IL-13* [59]. A single application of Etokimab was administered in a proof-of-concept study using 12 patients, and the improvement was observed to last for 140 days [60]. The G1k humanized anti-*IL-33* monoclonal antibody etokimab did not meet its primary objective in a 16-week phase II b clinical study on treating AD and is no longer being studied. Itepekimab (REGR3500), another *IL-33* inhibitor, proved insignificant in a phase II clinical trial [61].

#### 3.3.2. Adaptive Immune Response

The etiology of AD is excessively complex, reflected in the variability of its clinical phenomenology. The “march of the adaptive immune system,” which affects several pathways and offers a variety of therapeutic options, begins with the introduction of antigens [62]. Antigen presentation is the starting point for the fundamental “march that comprises the adaptive immune system”, which regulates several pathways and gives rise to various treatment options [32,63]. Apart from targeting Th2 immune response, which involves IgE, *IL-4*, *IL-5*, *IL-13*, *IL-31*, *IL-18*, *IL-37*, Janus kinase (*JAK*), *OX 40*, and *IL-4R* (a common receptor chain between *IL-13* and *IL-4*), there are other potential targets, including mediators associated with conditions like psoriasis, such as *IL-17*, *IL-36*, or *IL-22*, which are being explored in clinical studies [63,64]. In this section, we explore the key pathways of the adaptive immune response in AD patients and identify potential targets for treatment. We also provide a summary of the most significant findings from the latest clinical trials for each target, as detailed in Table 1.

- IL-4 and IL-13

The cytokines *IL-4* and *IL-13*, which are crucial in the pathophysiology of AD, appear to be the primary determinants of the Th2 immune axis. In mouse studies, they enhance *S. aureus* infections, resulting in pruritus, xerosis, inflammation, and an epidermal phenotype like AD [65]. *IL-13* and *IL-4* impair the barrier in AD skin and decrease the generation of proteins necessary for terminal differentiation [65].

The IgG4k *IL-13* antagonist tralokinumab prevents *IL-13* from interacting with *IL-13R1* and *IL-13R2*. Tralokinumab was FDA-approved in December 2021 for treating adult patients with moderate-to-severe AD. Three phase-III clinical trials remain ongoing, while six have already been completed. Conjunctivitis and upper respiratory tract infections were the most often-reported side effects [66]. Similar to this, lebrikizumab is an IgG4k monoclonal antibody that selectively binds to *IL-13*, blocking the heterodimerization of *IL-13Rα1*/*IL-4Rα* and the ensuing signaling, which ultimately stops the proliferation of AD [67,68].

- IL-5

*IL-5* causes eosinophils to migrate, which is important in relation to atopic diseases such as eosinophilic esophagitis and asthma. In AD patients, blood eosinophil levels are typically higher and appear to be correlated with the severity of this condition [69]. Patients with extrinsic AD and concomitant respiratory allergy illness had higher blood levels of eosinophils [70,71].

Benralizumab and mepolizumab inhibit the action of *IL-5* by inhibiting *IL5R*. Two phase-II studies, including on benralizumab, have been conducted; one is now complete, although the findings have not yet been made public [72,73]. A phase-II clinical trial involving the IgG1k *IL-5* inhibitor mepolizumab was eventually stopped because the criteria used and met were ineffective. Mepolizumab significantly reduced the quantity of peripheral blood eosinophils during 16 weeks of treatment. However, it did not meet the primary goals of clinical improvements [72].

- IL-31

Since this cytokine induces pruritus symptoms in AD patients, *IL-31* is known as the “itch cytokine” [74]. Activated macrophages, epidermal keratinocytes, eosinophils, basophils, DCs, and cutaneous peripheral nerves are among the immune cells that express *IL-31RA*, which is the target of the humanized monoclonal antibody nemolizumab. Through the inhibition of *IL-31RA*, AD inflammation and pruritus may be controlled [75,76]. Nemolizumab can fail to treat eczematous plaques as rapidly or effectively as conventional immunobiologics or *JAK* inhibitors despite reducing itching by blocking *IL-31* [77].

- JAK

For several cytokines, including interleukins like *IL-4*, *IL-13*, and *IL-31*, the *JAK/STAT* signaling pathway serves as a classical cascade. [78]. The complete blocking of *JAK* signaling, which is necessary for immune function and homeostasis, causes severe immunodeficiency [79]. *JAK* inhibitors exert limited and reversible effects by limiting competition to lower intracellular signal transmission, in contrast to biologics intended to change cytokine signaling pathways [80]. When cytokines, including *TSLP*, *IL-4*, *IL-13, IL-22*, and *IL-31*, bind to *JAK1* heterodimeric receptors in AD patients, this causes the receptors to be activated, which, in turn, causes Th2 cell differentiation and itching. Three *JAK* inhibitors, upadacitinib [81], abrocitinib [82], and baricitinib [83], have just acquired licenses for treating AD. Despite being biologics, which are large molecules administered parenterally, *JAK* inhibitors are small substances that may be used orally. Tyrosine kinase 2 (*TYK2*) and *JAK1/2/3* inhibitors, such as delgocitinib, have played a significant role in AD treatment. Following studies conducted by Nakagawa et al. [84] in Japan, delgocitinib 0.5% ointment received a license for AD treatment for adults and children, alongside a 0.25% ointment. Notable small molecules like ruxolitinib [85], cerdulatinib [86], and brepocitinib [87] function as selective *JAK* inhibitors, contributing to AD therapy.

Biological molecules, including dupilumab, tralokinumab, omalizumab, and nemolizumab, have demonstrated significant efficacy in regulating the *JAK*/*STAT* pathway and inhibiting *JAK*. This is particularly relevant as the *JAK*/*STAT* pathway encompasses interleukins such as *IL-4*, *IL-13*, and *IL-31* [88].

- OX40

*OX40* functions primarily as a costimulatory receptor and is a member of the *TNF* receptor superfamily. It expresses when T-cells, including effector and regulatory T-cells (Tregs), become stimulated. When there is inflammation, *TSLP* activates antigen-presenting cells, including endothelial cells and DCs, causing them to produce *OX40L* [89]. Additionally, it increases T-cell adhesion and migration and encourages and maintains the growth of Th2 central memory cells. By preventing further Th22 pathway activation after Th2 activation, blocking this receptor–ligand interaction may also promote the proliferation of Tregs and T-cell tolerance [90]. An anti-*OX40* monoclonal IgG1 antibody (GBR830) [91] passed phase IIa testing, and the findings are encouraging. Amlitelimab [92], an anti-*OX40L* monoclonal antibody, reduced IL-22 serum levels; however, there was no difference in IL-22 baseline serum levels between responders and nonresponders. Rocatinlimab (KHK4083) [93] is a monoclonal antibody against *OX40* that yielded positive results during a 16-week phase-II experiment. A novel selective phosphodiesterase 4 (PDE4) inhibitor, difamilast, is currently undergoing phase-3 trials. Difamilast achieved a significant milestone in 2021 by becoming the first PDE4 inhibitor to obtain manufacturing and marketing approval in Japan for treating both adult and pediatric AD patients, including those ≥2 years old [94].

- IL-4R

An excellent target is *IL-4R*, which encourages the signaling of *IL-4* and *IL-13*. The FDA has recently authorized the use of dupilumab, an IgG4 monoclonal antibody that explicitly targets *IL-4R*, for the treatment of AD for patients aged six months and older [95].

- IL-17A

In a pilot study (NCT02594098), secukinumab, an anti-*IL-17A* antibody presently used to treat plaque psoriasis, was explored with regard to AD treatment [96]. The findings showed that there were no discernible differences in clinical improvements (alterations in SCORAD and the EASI score from the beginning of the study) between the secukinumab group and the placebo group at week 16. These findings suggest that focusing on *IL-17* by itself is insufficient to effectively cure AD. Although another phase II-trial is complete, the results have not yet been released [97].

- IL-22

*IL-22* plays a vital role in the proliferation and downregulation of *FLG* in keratinocytes [98]. This seems an attractive therapeutic approach for AD treatment. Anti-*IL-22* fezakinumab can be used for the downregulation of *IL-22*. Furthermore, it was shown that fezakinumab-induced *IL-22* inhibition could cause the AD genomic profile to revert [99].

- IL-18 and IL-37

A recently identified anti-inflammatory member within the *IL-1* cytokine family is interleukin 37 (*IL-37*) [100]. Notably, children with AD exhibit significantly lower levels of *IL-37* in their skin barrier. It is crucial to highlight the relevance of the cytokine *IL-18* in relation to *IL-37* [101]. Compared to *IL-37*, *IL-18* provides a 50-fold-higher receptor affinity for *IL-18Rα* [102]. As biological drugs and *JAK* become more widely accessible, there is a strong focus on finding further treatments that directly adjust inflammatory processes and specifically target immune pathways or substances involved in AD’s development. Perhaps in the future, AD may be managed with therapies that stimulate cells to produce anti-inflammatory cytokines like *IL-37* or by administering synthetic anti-inflammatory cytokines [101,102].

**Table 1:** Current therapeutics for atopic dermatitis treatment [42,98,103,104,105].

Method	Mode of Application	Category	Therapeutic Agent	Name of Company	Target	Clinical Phase	ID of Clinical Trial
Microbiome modulation	Topical	Bacterial strain	B244	AO Biome	Nitric acid donor	IIb	NCT04490109
			FB-401	Forte Biosciences	TNFR activation and Bacterial substitution	IIB	NCT04504279
			ShA9	NIAID	Microbiota selected for transplant	I/Iia	NCT03151148
	Topical	Small molecule	Atx201/niclosamide	Union Therapeutics	Activity of protonophore	II	NCT04339985
			CLS-001/omiganan	Cutaneous Life Sciences	Improved cell membrane	II	NCT02456480
	Oral	Bacterial strain	EDP1815	Evelo	Regulation of systemic inflammation	Ib	NCT03733353
			STMC-103H	Siolta therapeutics	Using the microbiome to modulate immunity	Ib	NCT03819881
Target-innate immune response	Topical	Biologic	Tapinarof	Dermavant	*Ahr* agonist	IIB	NA
	Injection	Biologic	Tezepelumab	AstraZeneca	*TSLP*	IIA	NCT02525094
		Biologic	Etokimab	AnaptysBio	Interleukin-33	IIA	NCT03533751
		Biologic	REGN3500	Regeneron	Interleukin-33	IIA	NCT03738423
		Biologic	Astegolimab	Genentech	Interleukin-33	IIA	NCT03747575
		Biologic	MEDI3506	MedImmune	Interleukin-33	IIA	NCT04212169
		Biologic	Bermekimab	Janssen	Interleukin-1a	IIA	NCT03496974
		Biologic	Spesolimab	Bohringer Ingelheim	Interleukin-36R	IIA	NCT03822832
Target- Adaptive immune response	Injection	Biologic	CBP-201	Connect Biopharma	Interleukin-4Ra	IIB	NCT04444752
		Biologic	Dupilumab	Sanofi	Interleukin-4Ra	Approved	NCT03346434
		Biologic	ASLAN004	ASLAN	Interleukin-13Ra1	Ib	NCT04090229
		Biologic	Tralokinumab	LEO Pharma	Interleukin-13	Approved	NCT03526861
		Biologic	Lebrikizumab	Allmiral/Lilly	Interleukin-13	III	NCT04250350
		Biologic	Benralizumab	AstraZeneca	Interleukin-5Ra	IIA	NCT04605094
		Biologic	Omalizumab	Novartis	Immunoglobulin E	IIA	NCT02300701
		Biologic	FB825/anti-CεmX	LEO Pharma	Immunoglobulin E	IIA	NCT04413942
		Biologic	Fezakimuab	IIT	Interleukin-22	IIA	NCT01941537
		Biologic	Secukinumab	Novartis	Interleukin-17a	IIA	NCT03568136
		Biologic	Risankizumab	AbbVie	Interleukin-23	IIA	NCT03706040
	Injection	Biologic	Nemolizumab	Mitchga Syringes	Interleukin-31	Approved	NCT03921411
		Biologic	Mepolizumab	Nucala	Interleukin-5	II	NCT03055195
		Biologic	Amditelimab	Sanofi	*OX-40*	II	NCT03754309
		Biologic	Rocatinlimab	Kyowa Kirin	*OX-40*	II	NCT03703102
	Oral	Small molecule	Abrocitinib	Cibinqo	*JAK1*	III	NCT04345367
		Small molecule	Bacricitinib	Eli Lilly & Company	*JAK1*	III	NCT03334396
		Small molecule	Upadacitinib	Rinvoq	*JAK1*	III	NCT03569293
	Topical	Small molecule	Delgocitinib	Leo Pharma	*JAK1*	IIB	NCT03683719
		Small molecule	Ruxolitinib	Incyte Corp	*JAK1*	I	NCT03920852
		Small molecule	Cerdulatinib	AstraZeneca	*JAK1*	Ib	DMVT-502-1001
		Small molecule	Brepocitinib	Pfizer	*JAK1*	IIB	NCT0390382213

### 3.4. Treatment Strategies for AD and a Recently Developed Novel Drug Delivery System Based on Nanotechnology

**Figure 4:**
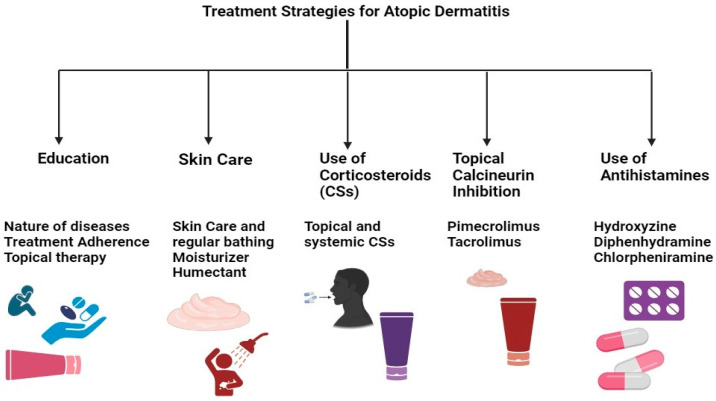
Multi-targeted treatment strategies for AD treatment.

The current research emphasizes therapeutic approaches that maximize formulation efficacy while minimizing adverse effects (Table 2) [106]. Treatment safety has been improved via numerous research efforts, including using novel drug delivery systems, combined therapy, and specific delivery systems (patch, liposome, nanoparticle, etc.) [107]. AD treatment plans center on lowering inflammation when necessary, healing skin, and eliminating itching. As a result, effective management and therapy make it feasible to apply a multi-targeted strategy that calls for caretaker and patient awareness (Figure 4). Additionally, it has been advised that taking care of the skin is necessary, entailing using topical calcineurin inhibitors (TCIs) for anti-inflammatory therapy, taking corticosteroids, and treating any skin infections. In cases of severity, systemic corticosteroids may also be used [108].

The treatment of AD encompasses skincare routines, topical solutions, systemic treatments, and preventive measures. Besides preventive measures and topical therapy, conventional systemic therapies include the use of drugs like cyclosporine, methotrexate, and azathioprine, alongside novel systemic medications such as biological agents and *JAK* inhibitors [34]. Notably, drugs targeting *IL-4*, *IL-13*, and *IL-33*, including dupilumab, and *JAK* inhibitors like baricitinib, abrocitinib, and upadacitinib have been approved or are pending approval in certain regions for moderate to severe AD. These treatments, especially *JAK* inhibitors, have demonstrated the ability to provide rapid itch relief and improve conditions, enhancing patients’ quality of life and presenting a generally favorable safety profile, though long-term safety data are still needed. Additionally, tralokinumab, a recently approved biological drug that specifically targets *IL-13*, has shown safety and efficacy, particularly when used with topical corticosteroids. Despite the effectiveness of these therapies, their high cost can limit access in some countries. The economic impact of these treatments and their affordability continue to be significant considerations, given the chronic nature of AD and its impact on quality of life. Measures such as EASI, the Dermatology Life Quality Index (DLQI), and the Pruritus/Itch Numeric Rating Scale are utilized to evaluate disease severity and treatment impact, underscoring the potential value of these therapies despite their expense. However, traditional biologics like rituximab, omalizumab, and ustekinumab are not recommended for treating AD, with mepolizumab being reserved for cases unresponsive to standard therapies [109].

Nanotechnology offers a safer and more effective method for treating various skin conditions, like AD, psoriasis, eczema, and cancer. Although nano-cosmetics are available, their potential for treating skin conditions still requires investigation. Nano-based drug delivery systems allow drugs to be precisely delivered to the skin with controlled release and diffusion, reducing off-target side effects. Nanoparticles can also overcome the skin’s natural barriers and poor drug solubility, enhancing drug delivery. Different nanoparticle formulations, including antibiotics, corticosteroids, herbal, synthetic, and combinations of these drugs, have been developed for treating AD through topical application [110,111,112].

In AD, ceramide levels drop, trans-epidermal water loss increases, and the skin barrier is compromised. As a result, surface engineering for vehicles has received much greater attention. API-loaded particles with the ideal charge, size, and surface transformation can treat skin conditions successfully [113,114]. Different nanosized systems, starting with liposomes, have been developed to improve the deep absorption of drugs into the skin. Polymeric particulates, nanoparticles made of lipids, dendrimers, and dendritic-core multi-shell nano transporters are some of the carriers that have been examined [115,116].

Lipid nanoparticles serve as carriers in place of liposomes, emulsions, and polymeric nanoparticles. The lipid NPs that showed the most potential for cutaneous application were solid lipid nanoparticles (SLNs) and nanostructured lipid carriers (NLCs). 

Yu et al. [117] developed chitosan-based nanoparticles (NPs) containing tacrolimus (FK506) and nicotinamide (NIC) as a hydrotropic agent. These NP exhibited high entrapment efficiency and stability in vitro. In vivo studies demonstrated sustained skin permeation for up to 24 hours in Sprague Dawley rats. BABL/c mice with DNCB-induced AD-like skin lesions treated with NPs showed lower dermatitis scores compared to the induced group. Additionally, measurements of ear skin thickness and splenic weight were conducted. In the DNCB group, the thickness was significantly higher than that for the treated mice, and spleen index elevation post-AD-induction suggested immune activation in the AD murine model. Treatment with NIC-CS-NPs loaded with FK506 suppressed activated immunity and decreased spleen index values, further reducing spleen index values compared to Protopic. The anti-inflammatory and immunosuppressive effects of NIC–CS–NPs were evident. In the histopathology of the DNCB-induced AD skin lesions, mast cell infiltration in DNCB-induced AD skin lesions was significantly decreased in the treatment group. Modified nano lipidic carrier-loaded gels showed noticeably improved release and penetration and greater bioavailability. Moisturizers that include nicotinamide (NIC) have successfully been used to treat AD [118].

Fan et al. [119] designed hyaluronic acid (HA)-decorated TAC-loaded NPs that were evaluated in vitro and found the drug’s effectiveness (there was a reduction in TEWL, skin integrity was maintained, and the histopathology results showed restoration of skin integrity), penetration, and release kinetics were sustained and controlled. These NPs had enhanced skin retention, anti-AD efficacy, and drug penetration capabilities. For the rationalized management of AD, NPs may be a useful therapeutic strategy, especially for adults and children with steroid phobia.

Niosomes are nonionic, drug-containing vesicular systems used for delivery developed using self-assembling hydrated surfactant monomers and used to improve drug solubility, bioavailability, and encapsulation effectiveness [120]. On the other hand, because of their vast surface area, extreme smallness, and excellent encapsulation effectiveness, polymeric nanoparticles are emerging as intriguing candidates for the topical administration of medicinal compounds. Chitosan is a polysaccharide made of chitin with a cationic charge used in tissue engineering and targeted medication delivery [121,122]. Due to the need for local medication delivery, it is more appropriate to create topical formulations for fatigued skin.

Betamethasone valerate (BMV)-loaded chitosan NPs adorned with HA were developed by Pandey and colleagues [123]. These particles possessed a 300 nm nanosize, a 58 mV positive zeta potential, an 86.56 entrapment efficiency, and a 34.72 loading capacity. Their drug dispersion and penetration efficiency were found to be suitable for treating AD. 

Using Carbopol^®^980 (Surfachem Group Ltd., West Yorkshire, UK) as a gel, Tessema and associates [124] investigated phytoceramides produced from oats that are included in nanocarriers, such as starch- and lecithin-based microemulsions and NPs. The delivery system exhibited essential physical-chemical characteristics. The microemulsion gel enhanced the absorption of oat ceramides into the deeper layers of the skin. Overall, the gel formulations proved effective in concentrating oat ceramides within the SC precisely where they were required. 

Espinoza et al. [125] developed a nanoemulsion of pioglitazone (PGZ) as a topical cream; PGZ, recognized for its anti-diabetic properties, demonstrated efficacy in modulating inflammatory responses, establishing itself as a potential therapeutic candidate for various skin diseases. The results obtained using an animal model demonstrated that the PGZ-loaded nanoemulsion suppressed inflammatory cytosines and reduced redness. This formulation has been proven to reduce the levels of inflammatory cytokines such as *IL-6*, *IL-1β*, and *TNF-α*. Histopathological studies have shown improved structural features of SC and reduced inflammatory cell penetration and thickness in the dermis. 

Alam et al. [126] designed a nanoemulsion for topical application by using clobetasol propionate (CP) as a therapeutic agent, employed for the treatment of skin disease, resulting in a significant inhibition of edema when compared to marketed cream (Glevate^®^, Dygen Pharma Distribution Corporation 1754E, Quezon City, Philippines); studies did not show evidence of irritation. These NCs, whose particle sizes typically range between 10 and 200 nm, deliver water-insoluble drugs to the skin’s deeper layers while reducing side effects by reducing dosage. 

When liposomes encapsulating BMV and diflucortolone valerate (DFV) were produced and added to chitosan gel, Eroglu et al. [127] observed extended drug retention, strong anti-inflammatory activity (evaluated using the carrageenan-induced paw edema method), decreased erythema, and quick lesion healing in a rat model. In vivo studies on Albino Wistar rats with DNFB-induced conditions showed that the corresponding treatment improved skin barrier function (evidenced by reduced TEWL), supported skin barrier recovery, and promoted hair regrowth. Visual skin examinations and histopathology revealed decreased mast cell activity, which is important in attenuating AD progression.

El-Menshawe et al. [128] designed thermally sensitive ethosomal gels with varying ratios of polymers to increase entrapment efficiency and vesicle deformability for topical nicotinamide (Vitamin B3) delivery. In induced rats (Wistar rat), the topical administration of optimized ethosomal gels diminished inflammation and corneocyte maturation, exhibiting an enhanced anti-inflammatory effect compared with Betaderm^®^ (TARO Pharmaceuticals Industries Ltd, Brampton, Canada) (0.1% Betamethasone valerate cream), decreases in TEWL, increased skin hydration, a reduction in histamine levels, and decreased IgE titers. 

Chauhan et al. [129] developed a transfersome that was loaded with glycyrrhizic acid (GA) and incorporated into hydrogel to evaluate its anti-inflammatory efficacy concerning the topical treatment of AD. In BALB/c mice induced with DNCB, the GA-Trans loaded gel induced the most significant reduction in scratching and erythema scores compared to other groups, showcasing the hydrogel formulation’s superior performance. The results highlight the substantial decline in in-vivo scratching and erythema scores with the GA-Trans loaded gel and underscore this formulation’s safety and efficacy with respect to addressing atopic dermatitis. 

Carreras et al. [130] pioneered the development of ultra-flexible lipid vesicles designed for the topical administration of cyclosporine A (CsA), an immunosuppressive medication utilized in the treatment of AD. The ability to cross the human epidermis was assessed in Franz diffusion cells, and these liposomal formulations effectively delivered CsA in the epidermis, according to the in vitro results. Nevertheless, in vivo studies are necessary to authenticate the anti-inflammatory effect. 

Kang et al. [131] investigated the use of thermosensitive solid lipid nanoparticles (NPs) for TAC delivery. These NPs showed high drug loading efficiency and achieved deeper skin penetration than commercial TAC ointments in an in vivo AD model, improving skin histopathology. Skin irritation tests conducted on rabbits using the Draize test revealed no irritation and only weak reddening after 24 hours of TAC-SLN application, indicating superior safety and effectiveness. 

Nagaich et al. [132] developed nanostructured lipid carriers (NLCs) for delivering betamethasone dipropionate (BMD). Comparing BMD-loaded NLC gel with traditional BMD gel on rat skin, the cited study showed that the NLC gel provided extended anti-inflammatory effects, suggesting its suitability as a once-daily treatment for AD. The NLC-based W/O ointment with BMD proved safe and effective for topical application, causing no skin inflammation or edema, minimizing systemic absorption side effects, and enhancing skin retention. 

Eiras et al. [133] developed NLCs that incorporated vitamin E (VE) into the hydrogel, and the results indicated the formulation’s adequate pharmaceutical properties. NLCs are well-known systems that demonstrate effectiveness in improving skin moisture and are recommended for cosmetic and dermatological usage. Regarding vitamin E and NLCs’ potential to increase skin hydration, it has been suggested that HG-NLCVE could be utilized in cosmetic applications (e.g., moisturizers and anti-aging) or treating dermatological illnesses such as AD. 

Ferreira et al. [134] developed Methotrexate (MTX)-loaded NLCs to enhance MTX bioavailability and dermal penetration. In vitro studies demonstrated an initial burst release followed by sustained release, reducing the need for frequent application. NLCs emerged as an innovative topical treatment for AD, holding promise in relation to improving drug safety, efficacy, and bioavailability with limited skin bioavailability, like MTX. 

Beclomethasone dipropionate (BDP) was used as a topical medication for treating AD via the formulation of nanocrystals, serving as an anti-inflammatory agent; the corresponding results revealed that when BDP-loaded nanocrystals were compared with Beclozone^®^ cream (Teva Pharmaceutical Industries, Parsippany, NJ, USA) (0.25% BDP cream), the optimized formulation showed higher drug deposition through mouse skin, despite reduced flux and low systemic exposure of the drug [135]. 

To address the issue of skin penetration, Pan et al. [136] formulated HA and cholesterol-based polymeric nanoparticles (NPs) and encapsulated TAC. In order to improve TAC and NIC’s solubility, a hydrotropic solution (20% *w*/*v*) was utilized. In vitro studies indicated that NIC, when incorporated into hyaluronic-acid/cholesterol-based nanoparticles (HA/Chol-based NPs), improves skin penetration and tacrolimus (TAC) deposition to a greater extent than Protopic^®^ 0.1% TAC ointment (Astellas Pharma Tech Co., Ltd., Toyama, Japan). Cell uptake experiments conducted using HaCaT cells and confocal laser scanning microscopy, using C6 as a fluorescent marker, showed that C6-loaded HA–Chol–NPs with NIC exhibited similar green fluorescence to HA–Chol–NPs alone. This suggests NIC does not affect the nanoparticles’ cellular uptake.

Boisgard et al. [137] introduced innovative semi-solid formulations (Avicel and Viscarin) subsequent to the development of fluorescent polylactic acid (PLA)-based NPs for anti-inflammatory purposes. The assessment of the NP suspension included the evaluation of spreadability and rheological behavior while maintaining the structure of the PLA-based NPs. This highlights the substantial potential of these formulations, incorporating PLA-based NPs, for targeted and topical SC administration with minimal systemic absorption. An in vivo skin irritation test (BALB/c mice) was performed, in which data on skin inflammation scoring, ear thickness, and histology were obtained to analyze inflammation severity. It was found there was no sign of inflammation, no visible ear skin inflammation, and no increase in ear thickness. The histological results showed a decrease in inflammatory cells upon filtration in the treatment group. 

Zabihi et al. [138] developed polylactide-co-glycerol (PLG) NPs for the topical delivery of TAC. After topical administration, the biodegradability of PLG was verified by incubating it with skin lysates, which lowers the possibility of toxicity and inflammation. Upon comparison with NPs that were developed using PLG to be marketed as Protopic^®^ (0.03% TAC cream), higher TAC levels were seen in the SC, epidermis, and dermis. Research conducted in vitro verified that TAC-loaded PLG-based NPs could reduce *IL-2* expression in a manner comparable to marketed cream. Additionally, *TSLP* showed an unanticipated considerable reduction with the topical administration of PLG-based NPs compared to the ointment.

A topical anhydrous formulation of NPs, which were PLGA-based and loaded with CsA, was developed by Badihi et al. [139]. Ex vivo studies showed that PLGA-based nanoparticles (NPs) enhance CsA penetration into deeper skin layers and significantly lower pro-inflammatory cytokine production, with *IL-6* and *IL-8* levels reduced by approximately 50%, indicating potent anti-inflammatory effects. In vivo studies conducted using an Ovalbumin-induced AD animal model revealed that the treated group had markedly lower levels of *INF-γ*, *IL-4*, and *IL-5*; reduced TEWL; and decreased OVA-IgE serum levels, alongside improvements in skin integrity and reduced skin thickness. These findings suggest that drug-loaded nanoparticles can be an effective alternative to systemic CsA delivery, offering deep skin penetration with minimal side effects, showcasing their potential as a topical drug delivery method.

BMV was encapsulated in PLGA or lecithin (LEC)/chitosan-based NPs by Ozcan et al. [140]. To achieve a suitable viscosity for easy skin application, NPs were added to chitosan gel formulations. The results indicate that the NP-loaded chitosan gel formulations enhanced BMV accumulation compared to marketed 0.1% BMV cream, prolonged skin residence, and minimized systemic toxicity. The NP-loaded chitosan gel provided greater anti-inflammatory effects than the cream, although it contained only 1/10 of the BMV concentration. The gel formulation exhibited good skin-whitening ability and does not cause any changes to the skin’s integrity after use.

Chitosan-based NPs were prepared by Hussain et al. [141] for the topical simultaneous administration of hydrocortisone (HC) and hydroxytyrosol (HT). These NPs can be delivered topically, show increased HC and HT accumulation in the skin, and lower the risk posed by corticosteroids. Mice treated with NPs exhibited improved in vivo control over TEWL, erythema intensity, dermatitis index, and skin thickness. The HC-NPs suppressed inflammatory cascades in serum and skin, including IgE, *IL-4*, *IL-5*, *IL-6*, *IL-13*, *IL-12p70*, *IFN-γ*, *TNF-α*, histamine release, prostaglandin-E2 expression, and *VEGF*-*α*. Fibroblast infiltration and elastic fiber fragmentation were blocked or significantly decreased at the cellular level. 

Barbosa et al. [142] formulated fucoidan/chitosan-based NPs for the topical application of MTX. The optimized formulation exhibited strong anti-inflammatory effects, evidenced by reduced levels of *IL-1β*, *IL-6*, and *TNF-α*, and was proven safe for topical use in treating AD with MTX-loaded NPs.

The new synthetic HNE inhibitor (ER143) synthesized by Marto et al. [143] was encapsulated in nanocapsules based on starch, which improved the inhibitor’s anti-inflammatory effects and exhibited controlled-release drug delivery, high drug retention and penetration in pig skin, and anti-inflammatory properties. Erythema and swelling were reduced by 98% after local use on mouse ears, and the ER143 lotion surpassed commercial lotions containing 0.1% hydrocortisone butyrate. Starch acts as a synergistic agent in anti-inflammatory actions.

To embalm desonide (DES), Rosa et al. [144] developed Eudragit^®^ RL100 nanocapsules to encapsulate desonide (DES), using either acai oil (AO) or medium-chain triglycerides (MCT) as the core. Both formulations were stable, with the AO-based one also offering UVA and UVC protection without phototoxicity. In vitro studies showed that DES-loaded NPs provided biphasic release, initially quick and then prolonged, making them an effective topical AD treatment. Similarly, Assem et al. [134] developed BDP-loaded polymeric micelles (PMs), which were incorporated into an HPMC hydrogel for topical application. Studies on CD-1 mouse skin showed superior drug permeation and deposition compared to Beclozone^®^ (Memphis Pharmaceuticals, Egypt) (0.25% BDP cream), with a histopathological analysis indicating that better AD skin healing was provided by the BDP-loaded PMs [145].

**Table 2:** Nanotechnology-based therapeutic approaches for atopic dermatitis treatment.

Delivery Agent	Formulation/Dosage Form	Technique Used	Inferences	References
Tacrolimus/Nicotinamide	Nanoparticles	Ionic gelation method	It enhances penetration through and into the skin’s layers, lowers treatment dosages, and acts as an adjuvant in the fight against AD.	[117]
Tacrolimus and hyaluronic acid	Chitosan-based nanoparticles	High-pressure homogenization solvent evaporation method	Their delivery system plays a significant role, which does not affect a patient’s preferences	[119]
Betamethasone and Hyaluronic acid	Chitosan-nanoparticles	High-pressure homogenization solvent evaporation method	A better-sustained release pattern and greater drug retention capacity were observed.	[123]
Oat-ceramides	Lecithin-based microemulsions and starch-based nanoparticles	Emulsification solvent evaporation method	The gel increased permeation of oat ceramides was deep into the skin.	[124]
Pioglitazone	Nanoemulsion	Water titration method	↑ stratum corneum hydration (SCH), ↑ suppression of inflammatory cytokines levels, redness reduction, ↓ dermis thickness, ↓ TEWL biocompatible with skin, and controlled release of the formulation were observed.	[125]
Clobetasol propionate	Nanoemulsion	NA	↓ edema inhibition and skin irritation.	[126]
Betamethasone valerate/Diflucortolone valerate	Liposomes/Nanoparticles	Thin-film hydration method/direct injection method	↑ edema inhibition, ↓ TEWL and erythema, and ↑ stratum corneum and epidermis retention.	[127]
Nicotinamide	Ethosome	Cold method	Results show that there was a decrease in IgE levels and inflammation. In vitro permeation results show that there was an increase in skin retention in rat models.	[128]
Glycyrrhizic acid	Transfersomes	Thin-film hydration method	The controlled release was followed by transfersomes for up to 24 h, ↓ in scratching and erythema score, and hematological parameters were normal.	[129]
Cyclosporine A	Multilamelar vesicles (transfersomes and ethosomes)	Thin-film hydration method	Ethosomes released the drug up to 24 h at controlled rate. If we compare this finding with the types of transfersomes, increases in flux and diffusion of CsA-loaded ethosomes were more concentrated than in other formulations.	[130]
Tacrolimus	Thermosensitive SLNs	Modified emulsification and low-temperature solidification.	In contrast to the reference product, it distributes more medications into deeper layers of skin and penetrates deeper into the epidermal layer.	[131]
Betamethasone valerate	NLCs	NA	When comparing drug-loaded NLC gel with BMV gel, a significant 2.59-fold increase in permeation, controlled release with non-Fickian diffusion, and a noteworthy increase in anti-inflammatory action were observed.	[132]
Vitamin E	NLCs	Hot high-pressure homogenization and ultrasound technique	In vitro studies showed that nanoparticles were biocompatible and non-irritant.	[133]
Methotrexate	NLCs	Hot ultrasonication method	The sustained release profile was shown by nanoparticles, which were non-toxicity and biocompatible.	[134]
Betamethasone dipropionate	Nanocrystal	Wet bead milling method	Ex vivo results show increases in drug accumulation in the skin layers and a decrease in flux.	[135]
Betamethasone dipropionate	Polymeric micelles	Thin-film hydration method	Polymeric micelles show increased skin retention of the drug and a ↓ the amount of drug permeated. Almost-complete skin healing was achieved before the conventional treatment.	[135]
Tacrolimus	NPs	Ultrasonication method	Results show increased skin retention and permeation.	[136]
Encapsulated fluorophore	NPs	Solvent diffusion method	No toxicity was seen over 8 days. Ex vivo results show decreases in permeation vs. poly lactic acid NPs suspension, ↑ inhibition of cell proliferation, and *IL-2* secretion.	[137]
Tacrolimus	NPs	NA	↑ in skin layer penetration; when the optimized formulation was compared to commercial treatment, an increase in inhibition of *IL-2* and *TSLP* was observed.	[138]
Cyclosporine A	NPs	NA	Results show an increase in the penetration of formulation into different deeper skin layers, preserved skin integrity, prevention of skin thickening, decreased swelling, and even in serum levels of *IFN-γ*, IgE, and *IL-4*.	[139]
Betamethasone valerate	NPs	Emulsion-diffusion-evaporation method	When a commercial cream was compared with the optimized NP loaded with BMV, it was found that there was a decrease in the skin thickness and blanching. Epidermis retention increased compared to that for the commercial treatment.	[140]
Hydrocortisone/Hydroxytyrosol	NPs	Ionic cross-linking method	There was an increase in skin retention and a decrease in flux, TEWL, intensity of erythema, skin thickness, and dermatitis index.	[141]
Methotrexate	NPs	Ultrasonication method	Results show great permeation of the drug into the skin.	[142]
ER143	Nanocapsules	Emulsion solvent evaporation method	There was an increase in erythema inhibition compared to conventional treatment, an ↑ in skin retention, and skin permeation.	[143]
Desonide	Nanocapsules	Interfacial deposition method	Slightly irritant, non-phototoxic, and shows a biphasic release profile	[144]
Auraptene	SLNs	Hot homogenization and ultrasound method	Improvement in anti-inflammatory properties and sustained release of drugs.	[146]
Nicotinamide	W/O/W multiple-emulsion and microemulsion	High agitation and incorporation	Multiple emulsions elicited no changes in the permeability of the skin	[147]
Mizolastine	PLGA-mPEG microparticles	O/W emulsification solvent evaporation method	Significant reduction in ear thickness and the dermatitis index as well as a suppression of the infiltration of cells related to inflammation and immunoglobulin E	[148]
Levocetirizine	Noisome and chitosan nanoparticles	Thin-film hydration method and ionic gelation method	Niosomes gel optimized had superior skin retention	[149]

Abbreviations: AD, Atopic dermatitis; W/O/W, Water/oil/water; SLNs, Solid lipid nanoparticles; O/W, Oil in water; NA, Not available; NLCs, Nanostructured lipid carriers; TEWL, Transepidermal water-loss; NPs, Polymeric nanoparticles; ER143, a human neutrophil elastase inhibitor. Symbols: ↑, increase; ↓, decrease.

## 4. FDA-Approved Medical Therapies for AD Treatment

Compared to traditional corticosteroids, topical calcineurin inhibitors (TCIs) are generally less efficacious and more irritating for the skin. TAC (Protopic) and Pimecrolimus (Elidel) were assigned a “Black Box Warning” from the Food and Drug Administration (FDA) Pediatrics Committee in February 2005 because of their propensity to induce cancer [150]. The FDA alerted doctors to the possibility of a connection between these malignancies and topical calcineurin inhibitors (lymphoma and skin cancer) in March 2005. However, recent studies have reported that TAC can be applied topically at a low concentration, ranging from 0.03% to 0.1%, to provide moderate patients with efficient therapy [151].

Another medication that is frequently used, particularly for adults, is methotrexate. It functions as an immunosuppressant drug, controlling the skin’s immune system (T cells), modifying immunological signals (cytokines), and calming down hyperactive inflammatory cells (neutrophils and monocytes). The immune system cells in the skin that cause Eczema are the focus of methotrexate. However, the only AD treatment recognized by most countries is cyclosporine [152,153].

A human monoclonal antibody, dupilumab (Dupixent), has been licensed for subcutaneous injection in severe AD patients as an *IL-4Ra* biologic target and an inhibitor to produce *IL-4/IL-13* [154,155]. Dupilumab reduced AD severity in Phase-II and -III randomized studies of adult patients [156] and has been approved by the FDA as a marketed treatment for AD [157,158]. 

Pfizer has gained FDA approval for cibinqo (abrocitinib), a supplementary New Drug Application (sNDA) for patients suffering from AD. The most recent approval expands the indications for cibinqo to teenagers aged 12 to 18 years, with conditions ranging from mild to moderate and severe AD and resistance or intolerance of other illegal substances. It was formerly authorized for the treatment of AD among people who were 18 years of age and above. An oral *JAK1* inhibitor called cibinqo controls several cytokines that have a significant role in the pathogenesis of AD. Interleukins, such as *IL-4*, *IL-5*, *IL-13*, *IL-22*, *IL-31*, and *TSLP*, are examples of cytokines. Data from the placebo-controlled, randomized Phase III JADE TEEN clinical trial support cibinqo label expansion. In middle-aged to teenage AD patients receiving background medication therapy, the trial compared the efficacy of 100 mg and 200 mg dosages of cibinqo to a placebo for these patients [159]. 

The FDA approved Danish Leo Pharma’s ADBRY (tralokinumab), ending dupixent’s monopoly on treating AD. Adults 18 years of age and above are administered immunization when topical corticosteroids alone cannot offer sufficient protection. For the medication tralokinumab, Leo paid AstraZeneca USD 444,115 million in 2016. This medication was initially created to treat asthma and inhibits the cytokine *IL-13*. The injectable monoclonal antibody dupixent, produced by Sanofi and Regeneron, was approved in 2017–2018 for AD treatment. It blocks the cytokines *IL-4* and *IL-13*. Adbry is the only FDA-approved biologic that explicitly binds *IL-13* and is the most targeted treatment for AD [160,161].

PDE4 is an enzymatic monoclonal antibody that helps immune cells release various cytokines. PDE-4 inhibition prevents the release of cytokines linked to AD inflammation. Currently, the FDA has approved a PDE4 inhibitor for the management of AD. It is advised to use the topical ointment crisaborole (Escriba^®^, Pfizer, New York, USA) for treating children and adults with mild to severe AD [162].

Eczema can be treated using topical *JAK* inhibitors [162,163,164]. The FDA has approved opzelura (ruxolitinib 1.5%, Incyte Biopharmaceuticals, North America, USA) cream as the first topical *JAK* inhibitor for the short-term and intermittent treatment of adolescents and adults (≥12 years) with mild-to-moderate AD [165].

## 5. Future Prospective

AD presents a multifaceted clinical profile, encompassing various overlapping factors in its pathophysiology. Unraveling the intricate pathophysiology of AD is vital for identifying novel therapeutic targets. The aim of devising a targeted therapy for AD is to minimize toxicity while improving barrier function and regulating inflammation and pruritus. There is a huge desire for novel therapies for adult and pediatric patients. Therefore, there is an unmet need for effective topical medicines that pose a low risk of adverse outcomes over time. Advanced treatments are vital for expanding options to help a wider range of diverse patients with AD. To enhance outcomes in both preclinical and clinical tests, the options for mechanism-based (*TNF-α*, *TLRs*, and *NF-κB*) or receptor-focused (such as *IL-4R*, *JAK*, and *IL-13R*) studies to help understand the underlying or fundamental causes of AD must be expanded.

Further research is imperative to create efficacious treatments for polarized immune pathways that remain unregulated by current anti-inflammatory agents. Developing validated biomarkers (for diagnosis, such as using NOS2/iNOS to monitor the severity of the disease, such as with *IL-18*, and for treatment, like with *IL-22* and *IL-18*) for identifying clinically significant immune pathways leading to AD is crucial for targeting specific mechanisms effectively. In the realm of AD research, scientific inquiry has paved the way for the identification of novel and efficacious treatments, paralleled by a deeper comprehension of this disease’s etiological factors. The recognition of biomarkers indicative of disease progression, akin to those identified in psoriasis, represents a methodological advance in assessing treatment effectiveness via molecular and histological regression. The evolution of biological therapies heralds a new era in dermatological treatment, with their integration into clinical practice and subsequent regulatory approval heralding the advent of tailored therapeutic strategies. This advancement not only promises to refine our understanding of AD’s pathophysiology across a diverse patient population but also underscores the potential of biological pharmaceuticals in the current research landscape as viable options for dermatitis management, aiming for treatments that are both less detrimental and more precisely targeted.

Furthermore, the exploration of innovative nanocarriers introduces a frontier in therapeutic delivery, merging with novel routes of administration to fine-tune nanoparticle systems specifically designed for AD management. The application of nanotechnology in dermatology is poised to address chronic inflammatory conditions effectively, with emerging nanomedicine techniques set to revolutionize the field. These advancements are anticipated to enable superior drug delivery mechanisms, offering enhanced therapeutic efficacy, minimized adverse effects, targeted drug delivery, and dosage reduction, thereby redefining the standards of clinical dermatological care.

## 6. Conclusions

The translational effort to convert our understanding from the pathophysiology of AD, including intriguing pathways and therapeutic targets, into drug discoveries facilitated the success of outstanding preclinical and clinical research. As a result, more treatment options are currently available for AD. Adopting a multidisciplinary approach enables more adaptive strategies and enhanced disease management, therapy compliance, and overall quality of life. The development of biotherapeutics targeting the pathways implicated in AD holds huge promise. Recent advances in understanding the biology of AD have led to better therapeutic outcomes. New therapies have resulted in significant advancements in the management of severe AD, which was previously resistant to traditional treatments. *JAK* inhibitors, initially employed for conditions like rheumatoid arthritis and myelofibrosis, offer an opportunity for the effective treatment of AD. These drugs, categorized as small molecules, are taken orally and do not trigger immune reactions, helping to overcome some of the drawbacks of biologics. Nanotechnology-based drug delivery systems may constitute a significant accomplishment regarding the treatment available to AD patients in the near future. In conclusion, this may be a new era in the treatment of AD with specific medications that are less/non-toxic and improve patient compliance.
